# Addressing Mental Health Needs After a Public Health Crisis in Southern Switzerland: Stakeholders’ Perspectives

**DOI:** 10.3389/ijph.2025.1608433

**Published:** 2025-08-27

**Authors:** Camilla Sculco, Manuela Barreca, Marco Meneguzzo, Emiliano Albanese

**Affiliations:** Università della Svizzera Italiana - Institute of Public Health, Lugano, Switzerland

**Keywords:** public health, stakeholder engagement, access to health services, COVID-19, mental health

## Abstract

**Objectives:**

This paper examines a stakeholder engagement initiative to develop public health recommendations for addressing mental health gaps after the COVID-19 pandemic in southern Switzerland. It explores local healthcare professionals’ experiences with the pandemic’s impact on mental health and service use.

**Methods:**

We used a stakeholder dialogue methodology based on participatory research. After mapping, we selected participants based on their power and interest on the subject. Data were gathered through two structured dialogues. We conducted thematic content analysis to identify and interpret patterns within dialogues. We used multi-step coding to assign codes to text segments, grouping similar patterns into themes and subthemes while ensuring consistency and exclusivity.

**Results:**

Thirty-two healthcare stakeholders from diverse sectors across urban and rural southern Switzerland participated in the dialogues. They emphasized flexible mental health service restructuring to address evolving patient needs and advocated for stronger prevention and promotion efforts, especially for vulnerable groups.

**Conclusion:**

Engaging local healthcare stakeholders turned up as an effective strategy to derive public health recommendations to improve mental health prevention and promotion along with the access and use of related services.

## Introduction

Ample evidence underscores the urgent and significant burden that COVID-19 has placed on the mental health (MH) of vulnerable populations living in Switzerland [1–3]. Studies revealed increased Common Mental Disorders (CMDs) such as depression and anxiety [4], post-traumatic stress disorder, and disturbed sleep [2] and their uneven occurrence in specific population groups, including young women, frail individuals, minorities [5] and people with low socio-economic status and educational background. Further, children and adolescents appeared to be significantly affected by the negative consequences of the pandemic on psychological wellbeing [6–8].

In Switzerland, the pandemic has not only affected the MH of the population but also the delivery of MH services, with changes in services provision varying significantly across regions [9]. In certain areas, MH services delivery was preserved with minor adjustments due to hygienic and physical distancing measures [9]. Other regions experienced disruption as services were re-organised due to shutdowns and closures of facilities such as rehabilitation programs, ambulatory clinics, and outreach services [9]. Face-to-face interactions and in-person visits were either halted, reduced, or rearranged, and closures entailed residential and day hospitals programs and restrictions on in-person outpatient services [9, 10]. The shutdown of services often made it impractical to establish and maintain therapeutic alliance, a critical factor in the success of psychiatric treatment [9].

The Swiss Learning Health System (SLHS) is a national platform for HC services that connects research, policies, and practices. It aims at fostering structured dialogue among stakeholders within the Swiss health system and promote the development and integration of research-based solutions to address current issues [11]. In this context, stakeholder participation and engagement is viewed as a means of co-producing knowledge through the development of recommendations [12].

Four years since the COVID-19 inception, the enduring MH needs of the population call for coordinated public health (PH) responses that account for the pre-pandemic and long-standing problems of scarce, unequal, and inefficient (mental) HC resources and services. Moreover, there is limited research on the experiences and perspectives of Swiss HC professionals regarding the impact of the pandemic on MH and the use of related services in Switzerland. Guided by the SLHS mandate, we launched a stakeholder engagement initiative to develop local-level recommendations for addressing unmet MH needs in the aftermath of the COVID-19 pandemic [13, 14]. In this paper, we present the results of the initiative, and we shortly discuss the implementation analysis, to address potential factors contributing to successful, failed, or delayed implementation of the recommendations.

## Methods

### Theoretical Framework

We used the SPICE (Sample; Phenomenon of interest; Context; Emphasis or focus) framework to define the research question and structuring the study design [15] (in Appendix). Additionally, we followed the Consolidated Criteria for Reporting Qualitative Research (COREQ) guidelines [16] to structure and format the paper and to ensure comprehensive reporting of qualitative method and findings (in Appendix). Our methodology is consistent with the WHO‐recognized guidelines [17] outlining a four-step process of identification, classification, assessment, and engagement.

We adopted a stakeholder dialogue methodology grounded in participatory research principles to explore stakeholders’ perspectives on MH needs and service gaps in southern Switzerland while gathering factual and qualitative data. Stakeholder engagement, as an extension of stakeholder theory (Freeman, 1984; Freeman et al., 2010) encompasses “*all activities aimed at fostering dialogue between an organization and its stakeholders, with the goal of providing a well-informed foundation for decision-making*” [18]. This method fosters collaboration among individuals leading to shared understanding and the co-production of recommendations. The methodology also aligns with the analytic-deliberative process devised by Deverka et. al., (2012), a framework used to examine the perspectives of multiple stakeholders by combining (1.) Analysis i.e., gathering stakeholders’ perspectives, preferences, and values; (2.) Deliberation i.e., engaging stakeholders in open dialogue to explore options and implications on the issue; (3.) Synthesis and decision-making i.e., integrating stakeholders’ input into decision-making processes, with mechanisms for feedback and iteration. Overall, the analytic-deliberative process ensures that diverse perspectives and values are thoroughly considered, thereby enhancing the transparency, legitimacy and effectiveness of decisions [19].

### Identification and Recruitment of Participants

**FIGURE 1:**
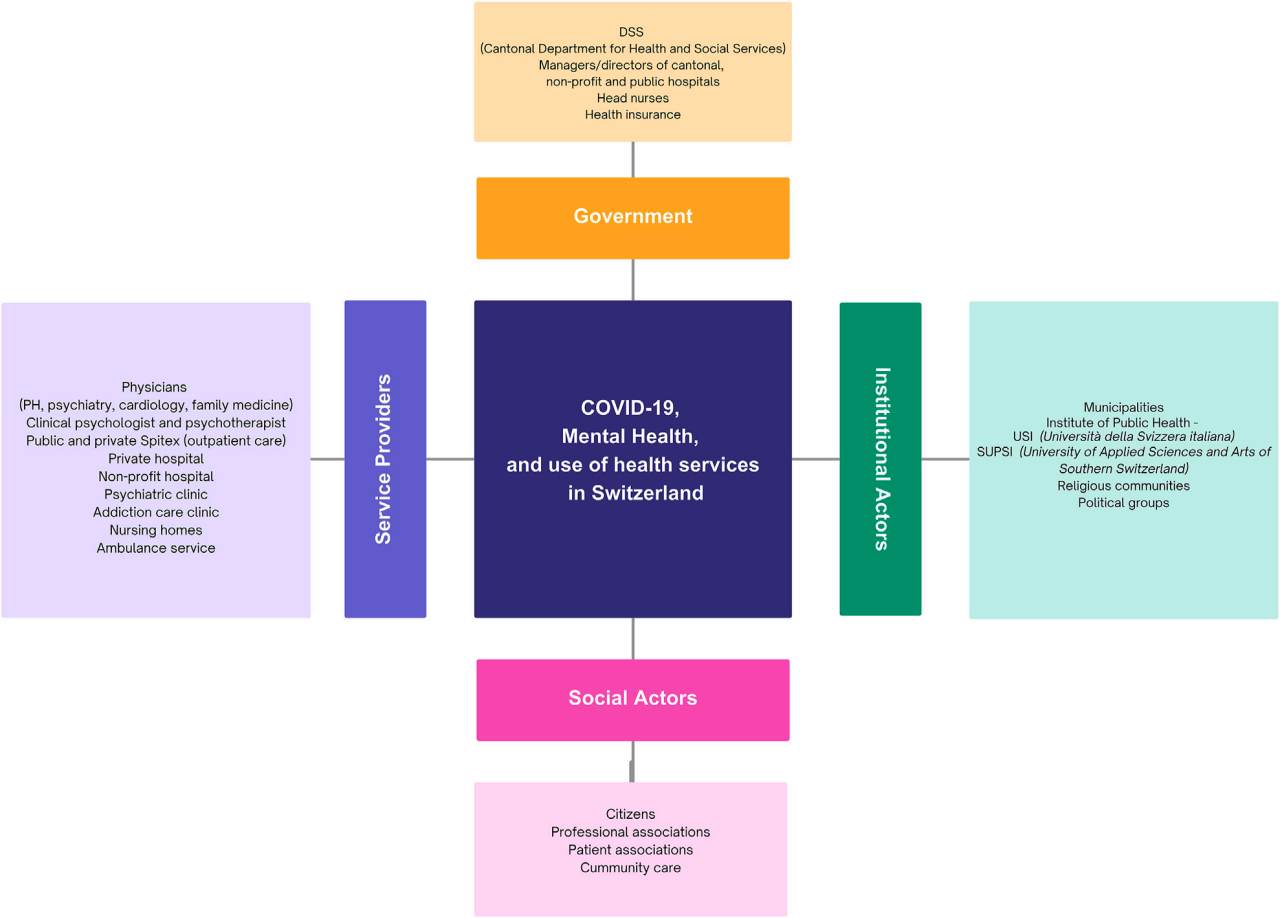
Stakeholder mapping. (Addressing Mental Health Needs After a Public Health Crisis in Southern Switzerland: Stakeholders’ Perspectives. Canton Ticino, Switzerland. 2024).

The identification and selection of local stakeholders was an important aspect of this study. This was completed with input and guidance from the senior author (MM) and after a careful mapping activity (Figure 1). The objective of this mapping phase was to identify and categorize all relevant actors systematically to ensure comprehensive representation throughout the dialogue and to identify potential gaps in the existing networks. Figure 1 presents a conceptual overview of all stakeholder categories identified in our mapping exercise, not the actual dialogue roster. Four actor groups are displayed: government, service providers, institutional actors, and social actors, each derived by inductive clustering and the prior knowledge of the researchers. Following, participants were selected based on their level of power (i.e., the ability to influence decisions, outcomes, and direction given by factors such as authority, expertise, resources), and their level of interest in the specific subject (i.e., the degree to which stakeholders are affected by or concerned about the issue) using the power versus interest grid [20]. This grid allows for the prioritization of stakeholders’ participation, ensuring that the most influential and affected individuals are actively involved: stakeholders with high power have significant control or authority over the issue, while those with low power have minimal influence. Similarly, stakeholders with high interest are directly impacted by the initiative or have a vested interest in its outcomes. Conversely, those with low interest may have little or no concern about the initiative’s progress or results [21].

Plotting stakeholders in the grid permits to prioritize their engagement and tailor communication strategies to build positive relationships and support through the initiative. In addition, we purposively selected stakeholders working in different settings (e.g., inpatient, outpatient, community care), in different sectors (i.e., public, non-profit, private), and in urban and rural regions of southern Switzerland. We tried to maximize inter-professionalism and to ensure different background and seniority. Selected stakeholders were contacted by email and informed about the initiative and its aim. Of the 45 stakeholders contacted 32 agreed to participate (71%). Lack of time or prior-planned commitments were amongst the reasons for declining participation.

### Conduction of the Dialogue and Data Collection

The investigator (CS) led the data collection with support of an observer who did not intervene in the dialogue and took notes of non-verbal communication among participants (MB). In April 2024, two distinct 2-h dialogues were conducted, each engaging different stakeholders: the first dialogue was held online (via MS Teams), the second took place in a dedicated meeting room at the Universita’ della Svizzera Italiana (USI). Both dialogues were conducted in a quiet and respectful atmosphere, facilitating interactions among participants without interruptions. The dialogues were conducted in Italian (the local language), and were audio recorded and transcribed verbatim by CS. Before conducting the dialogues, stakeholders were provided with a dialogue guide in form of a policy brief (PB) illustrating contextual information, the issue and preliminary evidence-based recommendations to tackle it [22]. In the dialogues, we asked stakeholders to share their experiences and perspectives on how the pandemic affected patients’ MH and the use of related services within their work contexts. Besides, we asked them to engage in discussion and agree on key PH recommendations for addressing unmet MH needs. Finally, we asked them to identify barriers and facilitators to the implementation of the recommendations. Discussions continued until no new arguments were mentioned.

### Data Analysis

The main investigator (CS) conducted data analysis with supervision by MB as thematic content analysis to identify and interpret patterns within the dialogues [23] he transcriptions of the dialogues were analysed in the original language and excerpts were translated only for the purpose of this study. The coding process was conducted as follows: first, the investigator carefully read the transcription to familiarize with the content. A first level of analysis was done by assigning codes to text segments that captured relevant aspects of the issue. Following this, codes with similar patterns were grouped into themes (i.e., working context, recommendation) and related subthemes (i.e., core element, barrier, facilitator). These themes and subthemes were then reviewed, combined, or divided, ensuring internal consistency and mutual exclusivity [24]. Finally, the themes and subthemes to retain were discussed with senior authors (EA, MB, MM).

## Results

### Characteristics of Stakeholders

A total of 32 stakeholders, representing various public and private organization in southern Switzerland (e.g., hospitals and clinics, nursing homes, long-term and community care centres, pharmaceutical companies, and health insurance) took part in the dialogue. The stakeholders were either directly or indirectly involved in MH care, and worked in different settings (e.g., inpatient, outpatient, community care), in both urban and rural areas in southern Switzerland (Table 1).

**TABLE 1:** Characteristics of stakeholders. (Addressing Mental Health Needs After a Public Health Crisis in Southern Switzerland: Stakeholders’ Perspectives. Canton Ticino, Switzerland. 2024).

Role	Setting	N	Sector	Region	Seniority
Clinical Psychologist and Psychotherapist	Private practice	1	Private	Urban	Junior
Physician	Public Health Psychiatry Cardiology Family medicine	2 1 1 1	Public Private Public Public	Urban Urban Urban Urban	Senior/Junior Senior Senior Junior
Head nurse	Cantonal hospital Nursing home Community care	3 3 1	Public Non-profit Non-profit	Urban/Rural Urban/Rural Rural	Senior Senior Senior
PhD candidate	University	1	Public	Urban	Senior
Parish priest	Parish church	1	Public	Rural	Middle
Case manager	Health insurance	1	Private	Urban	Senior
Manager/Director	Cantonal hospital Private hospital Psychiatric clinic Addiction care clinic Community care Nursing home Ambulance service Outpatient care Pharmaceutical company	1 1 2 1 5 1 1 2 1	Public Private Private Non-profit Private Non-profit Non-profit Non-profit Private	Urban Urban Urban Urban Urban/Rural Urban Urban/Rural Urban/Rural Urban/Rural	Senior Senior Senior Senior Senior Senior Senior Middle Senior

### Stakeholders’ Perspectives on the Impact of the Pandemic on Mental Health and the Use of Services

When referring to their working contexts in the aftermath of the COVID-19, stakeholders described increasing psychiatric visits and psychiatric hospitalizations, especially among children and adolescents, together with a growing demand for psychological therapy among young adults. Increasing urgent care interventions for MH problems from the ambulance services were also reported. Stakeholders observed that, the MH profile of patients and care users had evolved over time diversifying clinical expression, symptomatology, and complexity. Evolving profiles included: 1. Multimorbidity, such as common mental disorder combined with substance use disorder diagnosis. 2. Onset of psychological distress at an increasingly younger age. 3. Increased urgent psychiatric care. They deliberated that the process of profile evolution likely started several years before but that the COVID-19 pandemic exacerbated and accelerated it. Most physicians reported difficulties facing the emerging clinical expressions of psychological distress, and they reported uncertainty in clinical decisions about treatment and follow-up care, which contributed to greater referral of patients to specialists. Emerging changes and complexity required outpatient care services to ensure the provision of a multidisciplinary equipe comprising psychiatric nurses, social workers, and HC assistant. Finally, the psychotherapist perceived massive increase in requests of psychotherapy, difficulties in coping with the demand for consultations and long waiting lists.

### Public Health Recommendations

#### Considerations Towards Implementation of Recommendation: Barriers and Facilitators

Stakeholders deliberated on the following barriers towards implementation of the PH recommendations: first, Cantonal governments’ responsibility in local health service planning and the resulting differences in service provision across cantons. Specifically, stakeholders recognised cross-cantonal differences in mental disorder definition and in MH service provision. Second, stakeholders recognised that the fragmentation between inpatient and outpatient MH services can negatively affect both patients and HC professionals [25]. Similarly, stakeholders perceived the separation of financing system and insurance coverage between inpatient and outpatient MH services a challenge, in that differences disincentivize the quick transition of patients from acute to inpatient, and to the various outpatient care opportunities supported by mandatory health insurance [25]. Further, they recognised the relatively scarce resources allocated to hospital planning in psychiatric care. Similarly, they considered the technological development in psychiatric care lags compared to somatic care. All stakeholders recognised the shortage of HC professionals as a key barrier to meet the demand and expansion of services and to satisfy the needs of patients. Stakeholders devised the following facilitators towards implementation of the PH recommendations: first, they recognized the importance of political and institutional commitment. They deliberated that with telemedicine being part of the Health2030 policy strategy [26], Switzerland is progressing towards its integration. Second, stakeholders valued the integration of telemedicine consultations in the health insurances reimbursement process. Further, they deliberated on the positive effects stemming from the COVID-19 pandemic, that include increased attention and awareness on MH among the general population and among hospital staff. This ensures that risky situations are detected, reported, and forwarded to the competent HC professionals at an earlier stage. Finally, stakeholders considered that the introduction of the reimbursement of non-medical psychotherapy by the mandatory health insurance LAMal [27] has legitimated seeking MH support and has helped to reduce the stigma surrounding MH.

## Discussion

We explored stakeholders’ experiences and stances and derived PH recommendations to meet MH needs gaps while enhancing access and use of related services in the aftermath of the COVID-19 pandemic in southern Switzerland [Table 2 and 3]. Using a structured, theory-driven dialogue methodology, we uncovered the issue through the perspectives, values, and priorities of HC professionals. By exploring their experiences within their working context, we captured valuable insights into how the Swiss MH care system is responding to the pandemic’s impact and identified key elements for policymaking at the local level.

**TABLE 2:** First key public health recommendation agreed upon by stakeholders to address unmet mental health needs while enhancing access to services. Addressing Mental Health Needs After a Public Health Crisis in Southern Switzerland: Stakeholders’ Perspectives. Canton Ticino, Switzerland. 2024)

First key public health recommendation	Core elements
Re-structuring mental health services in a flexible way to cope with evolving profiles and increasing complexity of patients	Shifting the care paradigm by engaging governments and authorities, in line with a Whole-of-Society approach
	Personalizing care i.e., providing treatments that are tailored to the patients’ needs
	Investing in outreach services to engage patients who are resistant to be approached
	Enhance resources and expertise available in the Canton, by focusing on the concept of integrated care
	Intensify telemedicine to optimize healthcare resources

**TABLE 3:** Second key public health recommendation agreed upon by stakeholders to address unmet mental health needs while enhancing access to services. (Addressing Mental Health Needs After a Public Health Crisis in Southern Switzerland: Stakeholders’ Perspectives. Canton Ticino, Switzerland. 2024)

Second key public health recommendation	Core elements
Enhancing prevention and promotion of mental health and wellbeing both in the general population and for vulnerable population groups	Strengthening prevention and promotion of mental health and wellbeing for healthcare professionals by investing on awareness campaigns and support at the workplace
	Investing on education and public health campaigns by tailoring strategies to the specific needs of diverse population groups, reflecting on the messages to convey and the tools to employ
	Rethinking of awareness campaigns and promotion strategies with the support of everyday tools such as online platforms, podcasts, and social media

Overall, stakeholders conveyed that the pandemic has increased the demand for MH care, especially among the younger population. A similar finding was highlighted in other research conducted in Switzerland, which points out the psychological impact that the pandemic has had on the younger population. This is linked to changes in employment situations, restrictions related to school or training, increased family conflicts, and concerns about the future [28]. Likewise, the stakeholders observed that MH needs, and clinical expression of poor MH changed through the pandemic, though these changes were mainly attributed to social and anthropological factors rather than to the pandemic itself, and likely started several years before, they were likely exacerbated and accelerated by COVID-19. This finding is in line with a body of longitudinal literature in the field, which indicates a decline in the population’s MH across the years [29, 30]. Acknowledging the challenges of evolving profiles and increasing complexity of patients and care users, *flexibility* emerged as the keystone for restructuring and reorganizing MH services. Stakeholders interpreted flexibility as a shift in the care paradigm by engaging governments and authorities, in line with a Whole-of-Society approach [31]. The approach aims at fostering collaboration, governance, and action across sectors, while engaging non-state actors in collaboration with governments. This vision promotes the active involvement of frontline HC professionals in political decision-making and the co-creation of new policy initiatives. Further, the approach acknowledges the influence of the social determinants on health, and it recognizes that (mental) health is highly dependent on sectors beyond the HC [31]. The complexity of patients suggests the importance of implementing personalised and integrated care strategies by involving providers and professionals from different care sectors, while embracing a holistic and multidimensional approach [32]. Concerning this, stakeholders proposed the implementation of a structured triage system conducted by nursing staff to foster coordination between emergency, acute and primary care physicians. This nurse could assess the severity of symptoms, direct the patient to suitable resources, and coordinate necessary follow-up visits or treatments. Such a triage system would contain resources and cut waiting times, ultimately improving patients’ health outcomes [32]. A similar strategy is recommended in a wide body of literature that promotes the integrated care model to address the complex and emerging HC needs of the population, including concomitant physical, social, and MH impairments [33–36]. Further, stakeholders discussed the role of telemedicine in optimising resources in MH care and advocate for its increased use and for collective reflection on how to implement it best. Despite the limited technological development noticed in psychiatric care, the Swiss health system seems to be progressing towards the integration of telemedicine in traditional care, by acknowledging the Federal Council’s Health2030 policy strategy as a framework for action to overcome challenges in technological and digital transformation [26].

Stakeholders acknowledged the relevance of enhancing MH prevention and promotion campaigns both in the general population and for vulnerable population groups. Interestingly, when evoking the pandemic, stakeholders observed that HC professionals, typically regarded as a “strong” population group, were particularly vulnerable in terms of both physical and MH. Hence, they recommended strengthening MH services and support targeting the specific needs of HC professionals. Overall, stakeholders deemed the mental wellbeing of employees as an asset, and they reflected on the importance of MH prevention and promotion strategies for HC professionals by investing on MH awareness campaigns and support at workplace. The importance of prioritizing MH and wellbeing of HC workers has also been emphasized by Søvold and colleagues [37], who propose a set of policy recommendations and actions aimed at protecting HC professionals and reducing the risk of long-term psychological harm in the wake of COVID-19 and beyond.

Further, stakeholders suggested tailoring strategies to the specific needs of diverse population groups, reflecting on the messages to convey and the tools to employ when reaching them. They also proposed a rethinking of MH awareness campaigns and promotion strategies with the support of everyday tools such as online platforms, podcasts, and social media. Still, they stressed the importance of filtering quality messages and refining contents, highlighting the potential detriment of MH contents in social media. This observation aligns with the priority established in the international discourse to maximize the benefits of social media platforms while protecting users from the potential harms associated with them [38]. Besides, stakeholders contended that traditional PH campaigns are still insufficient due to their failure in addressing underlying cultural factors. What they advocate for are profound generational, cultural, and educational changes. Political and institutional commitment, which entails investing resources in the campaigns are also advocated for. Finally, stakeholders deliberated that the pandemic increased attention and awareness on MH among the general population and hospital staff. In connection with this, they considered that the introduction of the reimbursement of non-medical psychotherapy by the mandatory health insurance LAMal [27] has legitimated seeking MH support and helped to reduce the stigma surrounding MH.

### Limitations

Our study has some limitations. The qualitative design generated rich and multi-perspective information, but potential selection bias cannot be excluded in that we contacted a somewhat convenient sample of stakeholders that were easy to reach and that possibly know each other. While this may have contributed to reduce possible conflicts, opposing and diverging stances may have been less likely. Finally, no systematic independent coding of the dialogues was made but one researcher worked in autonomy, this is common in qualitative research that applies standard methods not prone to subjectivity.

### Policy Implications and Conclusions

The impact that COVID-19 has had on the population’s MH and access to services is a widely recognized issue at the international level [39]. Our project can offer models and lessons that can inform tailored approaches in other regions facing similar demographic or socio-economic challenges. Conducting research at the local level in Switzerland remains valuable internationally, as Switzerland is a high-income country with one of the most advanced HC systems in the world in terms of quality and accessibility [40]. Hence, Switzerland’s experience can serve as a valuable example, offering insights to inform and improve HC policies and practices in diverse contexts, particularly for policymakers aiming to improve and develop their systems at both the European and international levels. Besides, southern Switzerland has peculiarities that make it an interesting case study at the international level: it hosts a demographically older population, and its spatial and functional proximity to Northern Italy contributed to it being one of the first regions in Europe severely affected by the pandemic [41]. It also hosts a significant number of cross-border HC professionals [42].

When analysing techniques for implementing recommendations, it is important to consider aspects related with policy materialization, securing funding, preparing organizational conditions necessary for success, negotiating resources, and executing the policy recommendation [43]. Paying attention to these aspects permits to identify and possibly anticipate potential underlying causes of policy implementation failures or delays [43, 44]. Finally, attention should be paid to the critical issues to policy implementation such as resource diversion, goal misalignment arising from the engagement of multiple stakeholders, resistance to control over the dynamics and effectiveness of bureaucratic and administrative systems [45].

The COVID-19 pandemic highlighted the necessity for PH responses to meet new and established MH needs of the population while also addressing the long-standing issue of scarce, unequal, and inefficient resources [8]. The study offers novel perspectives on MH needs gaps recognized by local HC professionals in the aftermath of the pandemic. Engaging local stakeholders turned up as an effective strategy to derive PH recommendations to improve MH prevention and promotion along with the access and use of related services. The PH recommendations were directly incorporated into political decision-making; in fact, they were included in a policy brief, which was subsequently disseminated by the SLHS to support the development and integration of research-based solutions addressing current challenges [22]. A better understanding of the issue from the experiences and perspectives of local stakeholders provides insights on how the Swiss MH care system is addressing the repercussion of the pandemic. Further, it revealed aspects and guidelines to health policy making at the local level.

## References

[1] Aebi NJ Fink G Wyss K Schwenkglenks M Baenteli I Caviezel S Association of Different Restriction Levels with COVID-19-Related Distress and Mental Health in Somatic Inpatients: A Secondary Analysis of Swiss General Hospital Data. Front Psychiatry (2022) 13:872116. 10.3389/fpsyt.2022.872116 35592378 PMC9113023

[2] Ambrosetti J Macheret L Folliet A Wullschleger A Amerio A Aguglia A Impact of the COVID-19 Pandemic on Psychiatric Admissions to a Large Swiss Emergency Department: An Observational Study. Int J Environ Res Public Health (2021) 18(3):1174. 10.3390/ijerph18031174 33525740 PMC7908206

[3] Moser A Carlander M Wieser S Hammig O Puhan MA Hoglinger M. The COVID-19 Social Monitor Longitudinal Online Panel: Real-Time Monitoring of Social and Public Health Consequences of the COVID-19 Emergency in Switzerland. PLoS One (2020) 15(11):e0242129. 10.1371/journal.pone.0242129 33175906 PMC7657546

[4] Purtle J Nelson KL Counts NZ M Y. Population-Based Approaches to Mental Health: History, Strategies, and Evidence. Annu Rev Public Health (2020) 41:201–21. 10.1146/annurev-publhealth-040119-094247 31905323 PMC8896325

[5] Nadine T-J Eric B Henk MSV Monnay S Verloo H Head, development of Nursing Practices Unit, Valais hospital Perceived Stress, Trust, Safety and Severity of SARS-CoV-2 Infection Among Patients Discharged from Hospital During the COVID-19 Pandemic's First Wave: A Prems Survey. BMJ Open (2022) 12(6):e060559. 10.1136/bmjopen-2021-060559 35710249 PMC9207576

[6] Stocker D Schläpfer D Németh P Jäggi J Liechti L Künzi K. Der Einfluss Der COVID-19-Pandemie Auf Die Psychische Gesundheit Der Schweizer Bevölkerung Und Di psychiatrisch-psychotherapeutische Versorgung in Der Schweiz. Bern: Bundesamt für Gesundheit (2021).

[7] Werlen L Puhan MA Landolt MA Mohler-Kuo M. Mind the Treatment Gap: The Prevalence of Common Mental Disorder Symptoms, Risky Substance Use and Service Utilization Among Young Swiss Adults. BMC Public Health (2020) 20(1):1470. 10.1186/s12889-020-09577-6 32993605 PMC7526325

[8] Menges D Ballouz T Anagnostopoulos A Aschmann HE Domenghino A Fehr JS Burden of post-COVID-19 Syndrome and Implications for Healthcare Service Planning: A Population-based Cohort Study. PLoS One (2021) 16(7):e0254523. 10.1371/journal.pone.0254523 34252157 PMC8274847

[9] Richter D Bonsack C Burr C Gekle W Hepp U Kawohl W Therapeutic Alliance, Social Inclusion and Infection Control – towards pandemic-adapted Mental Healthcare Services in Switzerland. Swiss Arch Neurol Psychiatr Psychother (2021) 172. 10.4414/sanp.2021.03158

[10] Spigel R Lin JA Milliren CE Freizinger M Vitagliano JA Woods ER Access to Care and Worsening Eating Disorder Symptomatology in Youth During the COVID-19 Pandemic. J Eat Disord (2021) 9(1):69. 10.1186/s40337-021-00421-9 34112255 PMC8190763

[11] Swiss Learning Health System (2025). Available online at: https://www.slhs.ch/en/the-project/ (Accessed September 30, 2024).

[12] Greenwood M. Stakeholder Engagement: Beyond the Myth of Corporate Responsibility. J Business Ethics (2007) 74:315–27. 10.1007/s10551-007-9509-y

[13] Schuler D Tuch A Sturny I Peter C. Santé Psychique. In: Chiffres Clés Et Impact Du COVID-19. Neuchâtel: Observatoire Suisse De La Santé (2022).

[14] Schuler D Tuch A Sturny I Peter C. Santé Psychique. In: Chiffres Clés 2021. Neuchâtel: Observatoire Suisse De La Santé (2023).

[15] Booth A. Clear and Present Questions: Formulating Questions for Evidence Based Practice. Libr Hi Tech (2006) 24(3):355–68. 10.1108/07378830610692127

[16] Tong A Sainsbury P Craig J. Consolidated Criteria for Reporting Qualitative Research (COREQ): A 32-item Checklist for Interviews and Focus Groups. Int J Qual Health Care (2007) 19(6):349–57. 10.1093/intqhc/mzm042 17872937

[17] Schmeer K. Stakeholder Analysis Guidelines. (2001).

[18] O'Riordan L Fairbrass J. Managing CSR Stakeholder Engagement: A New Conceptual Framework. J Business Ethics (2014) 125:121–45. 10.1007/s10551-013-1913-x

[19] Deverka PA Lavallee DC Desai PJ Esmail LC Ramsey SD Veenstra DL Stakeholder Participation in Comparative Effectiveness Research: Defining a Framework for Effective Engagement. J Comp Eff Res (2012) 1(2):181–94. 10.2217/cer.12.7 22707880 PMC3371639

[20] Eden C Ackerman F. Power Interest Grid. Stakeholders Analysis and Management (1998). p. 349.

[21] Bryson JM. What to Do when Stakeholders Matter: Stakeholder Identification and Analysis Techniques. Public Management Rev (2004) 6(1):21–53. 10.1080/14719030410001675722

[22] Sculco C Meneguzzo M Albanese E. How Can Access to Mental Health Services in Switzerland Be Improved in the Aftermath of the COVID-19 Pandemic? Public Health Rev (2025) 46:1607659. 10.3389/phrs.2025.1607659 40673061 PMC12264636

[23] Braun V Clarke V. Using Thematic Analysis in Psychology. Qual Res Psychol (2006) 3:77–101. 10.1191/1478088706qp063oa

[24] Ta G. Applying Thematic Analysis Theory to Practice: A Researcher’s Experience. Conte Nurse (2005) 19:75–87. 10.5172/conu.19.1-2.75 16167437

[25] Stulz N Jorg R Reim-Gautier C Bonsack C Conus P Evans-Lacko S Mental Health Service Areas in Switzerland. Int J Methods Psychiatr Res (2023) 32(1):e1937. 10.1002/mpr.1937 35976617 PMC9976601

[26] FooP H. Health 2030 – The Federal Council’s Health Policy Strategy for the Period 2020–2030. Bern: Federal Office of Public Health (2019).

[27] Sculco C. Introduction of DRG System in Psychiatric Hospitals (2020). Available online at: https://eurohealthobservatory.who.int/monitors/health-systems-monitor/updates/hspm/switzerland-2015/introduction-of-drg-system-in-psychiatric-hospitals (Accessed September 30, 2024).

[28] De Quervain Dominique JF Amanda A Ehssan A Dorothée B Coynel D Gerhards C The Swiss Corona Stress Study. (2020).

[29] Ketchen LS Lattie EG Daniel DE. Increased Rates of Mental Health Service Utilization by U.S. College Students: 10-Year Population-Level Trends. Psychiatr Serv (2019) 70(1):60–3. 10.1176/appi.ps.201800332 30394183 PMC6408297

[30] Patalay Praveetha HGS Gage SH. Changes in Millennial Adolescent Mental Health and Health-Related Behaviours Over 10 Years: A Population Cohort Comparison Study. Int J Epidemiol (2019) 48(5):1650–64. 10.1093/ije/dyz006 30815691 PMC6904321

[31] Ortenzi F Marten R Valentine NB Kwamie A Rasanathan K. Whole of Government and Whole of Society Approaches: Call for Further Research to Improve Population Health and Health Equity. BMJ Glob Health (2022) 7(7):e009972. 10.1136/bmjgh-2022-009972 35906017 PMC9344990

[32] Luini C Calciolari S. Effect of Frailty on Healthcare Utilization: Policy Analysis and Recommendations to the Swiss Health System. Swiss Learn Health Syst (2023).

[33] Hendry A Taylor A Mercer S Knight P. Quarterly. Improving Outcomes Through Transforma Tional Health and Social Care Integration – The Scottish Experience. Healthcare (2016) 19:73–9. 10.12927/hcq.2016.24703 27700978

[34] Hendry A Vanhecke E Carriazo AM López-Samaniego L Espinosa JMSD Sezgin D Integrated Care Models for Managing and Preventing Frailty: A Systematic Review for the European Joint Action on Frailty Prevention (ADVANTAGE JA). Int J Integr Care (2019) 19:5–10. 31360661 PMC6581495

[35] Wodchis WP Dixon A Anderson GM Goodwin N. Integrating Care for Older People with Complex Needs: Key Insights and Lessons From a Seven-Country Cross-Case Analysis. Int J Integr Care (2015) 15(e021):e021. 10.5334/ijic.2249 26528096 PMC4628509

[36] Hoedemakers M Leijten Marie FR Looman W Czypionka T Kraus M al DH Integrated Care for Frail Elderly: A Qualitative Study of a Promising Approach in the Netherlands. Int J Integr Care (2019) 19(16):16. 10.5334/ijic.4626 31534444 PMC6729107

[37] Søvold LE Naslund JA Kousoulis AA Saxena S Qoronfleh MW Grobler C Prioritizing the Mental Health and Well-Being of Healthcare Workers: An Urgent Global Public Health Priority. Frtontiers in Public Health (2021) 9:679397. 10.3389/fpubh.2021.679397 34026720 PMC8137852

[38] Naslund JA Bondre A Torous J Aschbrenner KA. Social Media and Mental Health: Benefits, Risks, and Opportunities for Research and Practice. J Technology Behav Sci (2020) 5:245–57. 10.1007/s41347-020-00134-x 33415185 PMC7785056

[39] Sculco C Bano B Prina E al e Bartucz MB Barbui C Access and Use of General and Mental Health Services Before and During the COVID-19 Pandemic: A Systematic Review and meta-analysis. BMJ Open (2025) 15:e091342. 10.1136/bmjopen-2024-091342 40074252 PMC11904334

[40] Gutzeit A Dubsky P Matoori S Plümecke T Froehlich JM Bech-Hohenberger R Breast Cancer in Switzerland: A Comparison Between Organized-Screening Versus Opportunistic-Screening Cantons. ESMO Open (2024) 9(10):103712. 10.1016/j.esmoop.2024.103712 39321720 PMC11459637

[41] Goumenou M Sarigiannis D Tsatsakis A Anesti O Docea AO Petrakis D COVID‑19 in Northern Italy: An Integrative Overview of Factors Possibly Influencing the Sharp Increase of the Outbreak (Review). Mol Med Rep (2020) 22:20–32. 10.3892/mmr.2020.11079 32319647 PMC7248465

[42] Davoine E Salamin X. Cultural Influences in the Integration of Foreign Healthcare Workers. an Exploratory Study in the Swiss Health Sector. In: Internationalization and Organizations. New York: Routledge (2024).

[43] Michael C. Rational Techniques in Policy Analysis. USA: Heinemann Educational Books (1980).

[44] Pressman Jeffrey L. Implementation. United states: University of California Press (1984).

[45] Eugene B. The Implementation Game. In: What Happens After a Bill Becomes a Law. The MIT Press (1977).

